# Development and validation of a risk prediction model for chemical cystitis in patients with non-muscle-invasive bladder cancer undergoing intravesical instillation

**DOI:** 10.3389/or.2026.1791893

**Published:** 2026-03-16

**Authors:** Xinyu Yi, Zhaoyi Zhao, Jin Li

**Affiliations:** Department of Urology, Xiangtan Central Hospital, Xiangtan, China

**Keywords:** chemical cystitis, development and validation, intravesical instillation, non-muscle-invasive bladder cancer, predictive model

## Abstract

**Objective:**

To develop and validate a risk prediction model for Chemical cystitis in patients with non-muscle-invasive bladder cancer (NMIBC) undergoing intravesical instillation.

**Methods:**

This study retrospectively enrolled 225 patients with NMIBC who received intravesical instillation between January 2024 and January 2026. Predictive variables, including demographic characteristics, oncological features, medical history, treatment-related factors, and procedural anatomy, were collected. Feature selection was performed using the Least Absolute Shrinkage and Selection Operator (LASSO) regression from 18 candidate variables. A multivariable logistic regression model was constructed based on the selected variables and visualized as a risk prediction nomogram. The model’s performance was evaluated and validated using the Area Under the Curve (AUC), calibration curves, and Decision Curve Analysis (DCA) to assess discrimination, calibration, and clinical utility.

**Results:**

Five independent predictors were identified from the candidate variables through LASSO and multivariable logistic regression analysis: type of instillation agent, tumor multifocality, retention time of the agent, bladder capacity, and tumor grade. The predictive model demonstrated robust discriminative ability in both the training and validation cohorts, with AUC values of 0.840 and 0.868, respectively. Calibration curves showed high consistency between the predicted and observed risks, and DCA further confirmed the model’s positive net benefit in clinical decision-making.

**Conclusion:**

We successfully developed and validated a practical nomogram for the individualized prediction of Chemical cystitis risk in patients with NMIBC. This tool can assist clinicians in identifying high-risk patients prior to treatment, thereby enabling more targeted monitoring and preventive strategies. This study is limited by its single-center retrospective design, and external prospective validation is warranted.

## 1 Introduction

Bladder cancer (BC) remains one of the most prevalent urological malignancies worldwide. According to the latest epidemiological data, BC is the ninth most common cancer globally, with approximately 614,000 new cases and 220,000 deaths reported in 2022 (1). Histopathologically, BC is categorized into non-muscle-invasive bladder cancer (NMIBC) and muscle-invasive bladder cancer (MIBC) based on the depth of tumor invasion (2). For patients with NMIBC, intravesical instillation therapy following transurethral resection of bladder tumor (TURBT) is the established standard for adjuvant treatment. Immediate post-operative instillation effectively eradicates disseminated tumor cells and residual lesions, thereby reducing the risk of early recurrence. For intermediate- and high-risk patients, induction therapy combined with maintenance instillation—tailored to individual risk stratification—further improves clinical prognosis (3).

However, the prolonged duration of intravesical therapy and repeated exposure to chemotherapeutic agents can induce mucosal damage, leading to the development of Chemical cystitis. As intravesical therapy becomes more widely implemented, the incidence of such complications has progressively increased (4). The clinical manifestations of Chemical cystitis, including frequency, urgency, dysuria, and hematuria, are non-specific and closely mimic those of common urinary tract infections, frequently leading to misdiagnosis or delayed intervention. If not managed promptly, Chemical cystitis may progress to bladder fibrosis, resulting in reduced bladder capacity and permanent functional impairment, which severely compromises the patient’s quality of life (5). Despite the increasing clinical recognition of this complication, a systematic and individualized risk prediction tool to identify high-risk patients is currently lacking (6).

Consequently, this study aims to identify independent predictive factors for Chemical cystitis in NMIBC patients undergoing intravesical instillation through a retrospective analysis. Furthermore, we developed and validated a visual risk prediction nomogram. This tool is designed to assist clinicians in pre-treatment risk assessment and the formulation of personalized monitoring and preventive strategies, ultimately enhancing treatment safety and patient quality of life.

## 2 Materials and methods

### 2.1 Study population

This single-center retrospective study consecutively enrolled 225 patients diagnosed with non-muscle-invasive bladder cancer (NMIBC) who underwent transurethral resection of bladder tumor (TURBT) followed by adjuvant intravesical instillation at Xiangtan Central Hospital between January 2024 and January 2026. This study was approved by the Ethics Committee of Xiangtan Central Hospital. All procedures and data collection were conducted in accordance with the principles of the Declaration of Helsinki, and written informed consent was obtained from all participants.

### 2.2 Inclusion and exclusion criteria

The inclusion criteria were: 1. age 18–80 years (The upper age limit was set because very advanced age is frequently associated with a higher burden of comorbidities and frailty, which may confound symptom-based endpoints and tolerance to intravesical instillation); 2. histopathologically confirmed NMIBC (Stage Ta, T1, or Tis); 3. completion of a full course of intravesical therapy (induction phase or induction combined with maintenance phase) following TURBT; 4. complete clinical and follow-up data. The exclusion criteria were: 1. pre-existing chronic cystitis due to non-chemical factors (e.g., interstitial cystitis, radiation cystitis); 2. concurrent active urinary tract infection (UTI) or other acute pelvic inflammatory diseases; 3. severe systemic diseases potentially affecting prognosis (e.g., uncontrolled heart failure, hepatic or renal failure); 4. presence of severe urethral stricture or neurogenic bladder; 5. missing follow-up information.

### 2.3 Cohort allocation

Using a computer-generated random number method, all eligible patients were randomly assigned to the training cohort and the internal validation cohort in a 7:3 ratio. These cohorts were used for risk prediction model construction and internal validation, respectively.

### 2.4 Data collection

The parameters collected included: Demographic characteristics: Gender, age, body mass index (BMI), smoking history, and Eastern Cooperative Oncology Group (ECOG) performance status.

Oncological features: Tumor stage, tumor grade, maximum tumor diameter, tumor multifocality, and prior recurrence history.

Medical history: Hypertension, coronary heart disease, and diabetes. Treatment-related factors: Type of instillation agent and dosage. Procedural and anatomical factors: Surgical approach, agent retention time, and bladder capacity.

Data were extracted from the hospital electronic medical record system, including inpatient and outpatient charts, operative/anesthesia records, pathology reports, medication and intravesical instillation logs, and follow-up documentation. Data were extracted by trained clinicians using a standardized form.

### 2.5 Definitions and treatment protocols

ECOG Performance Status: Scored from 0 to 5, where 0 represents fully active and 5 represents death.

Chemical cystitis: Defined according to the 2023 EAU guidelines as new-onset or significantly worsened lower urinary tract symptoms (LUTS) following instillation (including at least two of the following: frequency, urgency, dysuria, suprapubic pain, or gross/microscopic hematuria). Diagnosis further required: 1. sterile pyuria or hematuria on urinalysis; 2. negative mid-stream urine culture to exclude bacterial infection; 3. symptoms requiring clinical intervention (e.g., delayed instillation, use of antispasmodics, analgesics, or bladder mucosal protectants).

Instillation Procedure: Standard aseptic techniques were employed. Following bladder emptying via catheterization, prescribed doses of agents—including immunomodulators (BCG) and chemotherapeutic agents (e.g., pirarubicin, gemcitabine)—dissolved in 20–50 mL of normal saline were slowly instilled into the bladder. The agent was retained for a specific duration before spontaneous voiding.

### 2.6 Statistical analysis

Data analysis was performed using SPSS version 26.0 and R software. Continuous variables were expressed as mean ± standard deviation (SD). Categorical variables were compared using the Chi-square test or Fisher’s exact test, while continuous variables were compared using the Wilcoxon rank-sum test. A *P*-value < 0.05 was considered statistically significant.

Candidate variables from the training cohort were entered into a Least Absolute Shrinkage and Selection Operator (LASSO) regression model for dimensionality reduction and feature selection. To reduce the risk of overfitting, predictor selection was performed using LASSO regression with 10-fold cross-validation to identify variables with non-zero coefficients and shrink estimates. Model performance was then evaluated in an internal validation cohort created by a pre-specified 7:3 split, assessing discrimination and calibration. Subsequently, independent risk factors associated with Chemical cystitis were identified via multivariable logistic regression analysis to construct a nomogram. The model’s performance was evaluated using Receiver Operating Characteristic (ROC) curves and the Area Under the Curve (AUC) for sensitivity and specificity. Calibration curves and Decision Curve Analysis (DCA) were performed to assess predictive accuracy and clinical net benefit. Finally, the validation cohort was used to internally validate the performance of the nomogram.

## 3 Results

**FIGURE 1:**
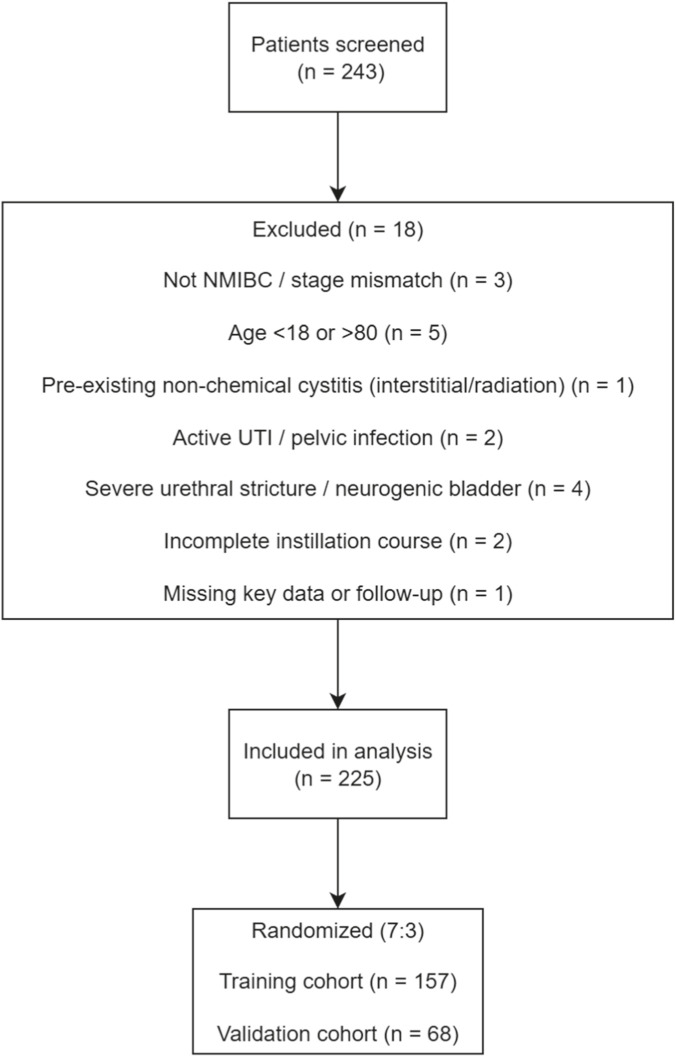
Flowchart of patient selection and cohort allocation. Patients with NMIBC undergoing TURBT followed by intravesical instillation between January 2024 and January 2026 were screened. After applying inclusion and exclusion criteria (with reasons), eligible patients were randomly allocated into the training cohort (70%) and internal validation cohort (30%).

The patient selection process is summarized in Figure 1.

A total of 225 patients were enrolled in this study, of whom 70 (31.1%) developed Chemical cystitis. The study population included 145 males and 80 females, with a mean age of 67.8 ± 9.2 years and a mean BMI of 24.1 ± 3.2. Following the 7:3 randomization, 157 patients were assigned to the training cohort, while 68 patients were assigned to the validation cohort. The clinical characteristics of patients in both cohorts are summarized in Table 1. There were no statistically significant differences in the distribution of any baseline characteristics between the training and validation cohorts (*P* > 0.05), demonstrating good comparability between the two groups.

**TABLE 1:** Comparison of characteristics between the training and validation cohort.

Variable	All cases (n = 225)	Training cohort (n = 157)	Validation cohort (n = 68)	*P* value
Age (years)	67.8 ± 9.2	67.9 ± 9.4	67.5 ± 8.8	0.765
Male sex, n (%)	145 (64.4%)	102 (65.0%)	43 (63.2%)	0.796
BMI (kg/m^2^)	24.1 ± 3.2	24.1 ± 3.3	24.2 ± 3.0	0.832
ECOG score, n (%)	​	​	​	0.942
0	75 (33.3%)	52 (33.1%)	23 (33.8%)	​
1	95 (42.2%)	66 (42.0%)	29 (42.6%)	​
2	43 (19.1%)	30 (19.1%)	13 (19.1%)	​
≥3	12 (5.3%)	9 (5.7%)	3 (4.4%)	​
Smoking history, n (%)	68 (30.2%)	48 (30.6%)	20 (29.4%)	0.865
T stage, n (%)	​	​	​	0.975
Ta	119 (52.9%)	83 (52.9%)	36 (52.9%)	​
T1	82 (36.4%)	57 (36.3%)	25 (36.8%)	​
Tis	24 (10.7%)	17 (10.8%)	7 (10.3%)	​
Tumor grade, n (%)	​	​	​	0.941
Low grade	92 (40.9%)	64 (40.8%)	28 (41.2%)	​
High grade	133 (59.1%)	93 (59.2%)	40 (58.8%)	​
Tumor multiplicity	​	​	​	0.925
Single	140 (62.2%)	97 (61.8%)	43 (63.2%)	​
Multiple	85 (37.8%)	60 (38.2%)	25 (36.8%)	Multiple
Tumor size (cm)	1.82 ± 0.95	1.81 ± 0.98	1.84 ± 0.89	0.830
Hypertension	110 (48.9%)	77 (49.0%)	33 (48.5%)	0.943
Diabetes	55 (24.4%)	39 (24.8%)	16 (23.5%)	0.835
Intravesical agent	​	​	​	0.956
BCG	48 (21.3%)	34 (21.7%)	14 (20.6%)	​
Pirarubicin (THP)	105 (46.7%)	73 (46.5%)	32 (47.1%)	​
Gemcitabine	72 (32.0%)	50 (31.8%)	22 (32.4%)	​
Retention time (min)	88.5 ± 42.6	89.2 ± 43.1	86.8 ± 41.5	0.698
Bladder capacity (mL)	325.4 ± 88.2	324.1 ± 90.5	328.4 ± 83.4	0.738
Cystitis	70 (31.1%)	49 (31.2%)	21 (30.9%)	0.960

**FIGURE 2:**
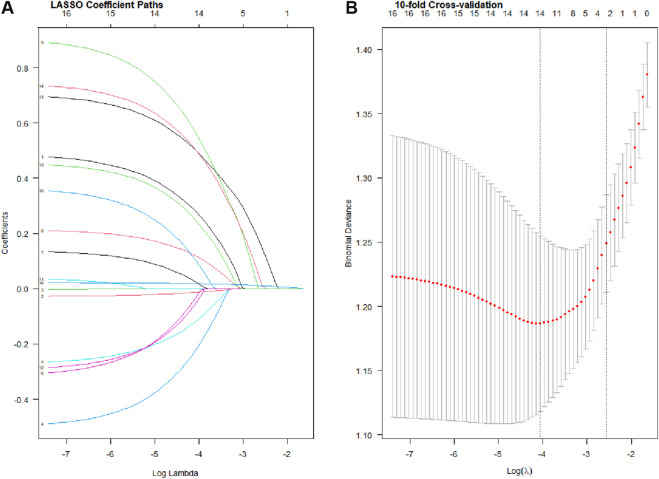
Profiles of the LASSO coefficients for the 18 candidate variables. Features selection by LASSO **(A)**. Ten-fold cross-validation for tuning parameter selection in the LASSO model **(B)**.

In the training cohort, all 18 candidate variables were initially analyzed using the Least Absolute Shrinkage and Selection Operator (LASSO) regression to prevent overfitting and address potential multicollinearity. Based on the optimal lambda (λ) value determined by the minimum criteria, five potential predictors with non-zero coefficients were derived from the original 18 variables (Figure 2). These five independent predictive factors included: type of instillation agent, tumor multifocality, agent retention time, bladder capacity, and tumor grade.

Collinearity diagnostics yielded a mean Variance Inflation Factor (VIF) of 1.16, indicating no significant multicollinearity among the candidate variables. In multivariable logistic regression, intravesical agent type (OR 3.58, 95% CI 1.49–8.61, *P* = 0.004), tumor multifocality (OR 2.15, 95% CI 1.12–4.12, *P* = 0.021), retention time (OR 1.26, 95% CI 1.10–1.45, *P* = 0.001), bladder capacity (OR 0.81, 95% CI 0.70–0.94, *P* = 0.006), and tumor grade (OR 2.43, 95% CI 1.25–4.72, *P* = 0.009) were independently associated with chemical cystitis (Table 2).

**TABLE 2:** Multivariable logistic regression analysis of independent predictors of Chemical cystitis in the training cohort.

Variable	VIF	OR	95% CI	*P* value
Intravesical agent	1.25	3.58	1.49–8.61	0.004
Tumor multiplicity	1.12	2.15	1.12–4.12	0.021
Retention time	1.18	1.26	1.10–1.45	0.001
Bladder capacity	1.14	0.81	0.70–0.94	0.006
Tumor grade	1.09	2.43	1.25–4.72	0.009

**FIGURE 3:**
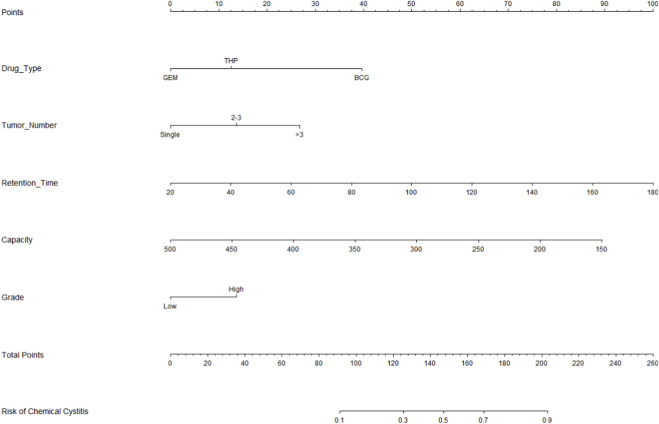
A nomogram for predicting Chemical cystitis.

A visualized nomogram for predicting Chemical cystitis risk in NMIBC patients was constructed by integrating the five independent predictors: type of instillation agent, tumor multifocality, agent retention time, bladder capacity, and tumor grade (Figure 3).

**FIGURE 4:**
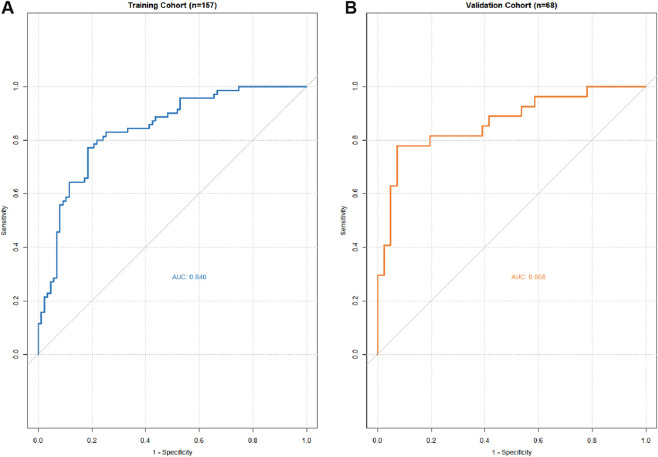
AUC of the ROC curve in training cohort **(A)** and validation cohort **(B)**.

ROC curve analysis demonstrated excellent discriminative performance of the prediction model, with Area Under the Curve (AUC) values of 0.840 in the training cohort and 0.868 in the validation cohort (Figure 4). These results indicate that the nomogram can accurately distinguish high-risk NMIBC patients susceptible to Chemical cystitis.

**FIGURE 5:**
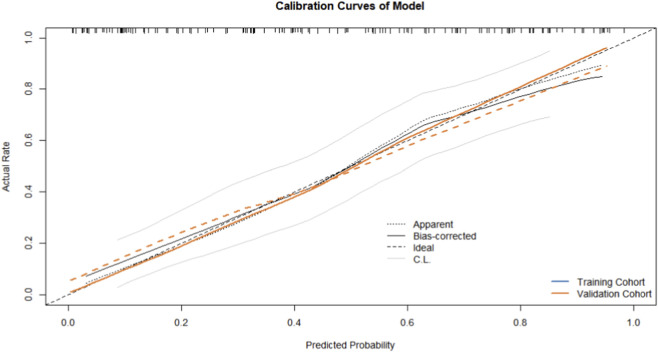
A calibration curve for predicting Chemical cystitis in training cohort and validation cohort.

**FIGURE 6:**
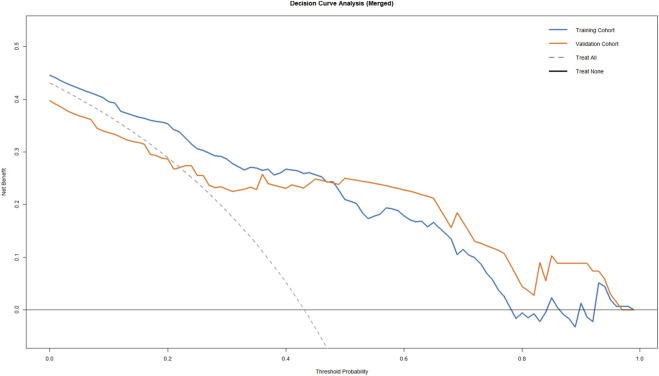
Decision curve analysis (DCA) for predicting chemical cystitis in the training and validation cohorts.

The calibration curves for both cohorts showed a high degree of proximity between the predicted and observed probabilities (Figure 5), indicating excellent consistency. Furthermore, Decision Curve Analysis (DCA) demonstrated that using this model for clinical decision-making provides a higher net benefit, confirming its substantial clinical utility (Figure 6).

## 4 Discussion

Non-muscle-invasive bladder cancer (NMIBC) is a prevalent urological malignancy, for which post-operative prophylactic intravesical instillation remains the standard strategy to reduce recurrence (2, 3). However, Chemical cystitis (CC), the most prominent local complication, significantly increases patient suffering and the burden on healthcare systems (7). In the absence of specific preventive measures, approximately 30%–45% of patients experience varying degrees of inflammatory reactions, leading to the interruption of treatment protocols (8, 9). In the current study, the overall incidence of CC was 31.1%, consistent with existing literature.

Despite the high incidence of CC during instillation, no predictive model has previously been available to quantify this risk. This study developed and validated a nomogram to predict CC risk in NMIBC patients based on five predictors: type of instillation agent, tumor multifocality, retention time, bladder capacity, and tumor grade. The AUC values of 0.840 and 0.868 in the training and validation cohorts, respectively, demonstrate the robust predictive performance of the model, which can facilitate personalized counseling and instillation management.

Tumor burden is widely utilized in the management of urinary tract tumors and has been proven as a predictor of clinical outcomes for various surgical and adjuvant therapies (2). Recently, tumor burden was also confirmed to be closely associated with instillation tolerance (10). Tumor multifocality and grade are commonly used to quantify this burden. Observational studies suggest that a higher number of tumors detected via imaging or endoscopy correlates with larger surgical wounds, increasing the risk of agents penetrating the interstitium through damaged mucosa (11). Furthermore, high-grade tumors exhibit more pronounced disturbances in epithelial polarity, further weakening the mucosal barrier. Our model confirms that tumor multifocality significantly influences CC occurrence (OR = 2.15, 95% CI:1.12–4.12, *P* = 0.021), as does tumor grade (OR = 2.43,95% CI:1.25–4.72, *P* = 0.009), identifying tumor burden as a critical predictor.

The design of the instillation protocol—specifically the agent type and retention time—acts as a direct physical trigger for CC. BCG, pirarubicin (THP), and gemcitabine (GEM) are the most frequently used agents. Due to differing pharmacological properties, THP typically exhibits higher mucosal irritation compared to GEM. Sylvester et al. found that BCG-induced bladder irritation rates were significantly higher than those of chemotherapeutic agents (12). Similarly, prolonged drug retention increases the probability of chemical injury. We identified retention time as a key predictor (OR = 1.26, *P* = 0.001), as extended durations (e.g., >90 min) may lead to sustained damage to the glycosaminoglycan (GAG) layer.

Bladder capacity was identified as the sole protective factor in this study (OR = 0.81, *P* = 0.006), reflecting the importance of physiological compensatory capacity in preventing CC. While systemic indicators like serum uric acid or creatinine are effective in some models, bladder capacity has a more direct diluting effect on the concentration of the instillation agent (13). A larger capacity not only reduces the drug dose per unit area but also alleviates intravesical high pressure during instillation. Therefore, assessing and improving bladder capacity may represent a potential measure to mitigate CC.

The nomogram provides an easy-to-use tool for estimating an individual patient’s risk of chemical cystitis at the time of intravesical instillation planning. By incorporating readily available clinical variables (instillation agent type, tumor multifocality, planned retention time, bladder capacity, and tumor grade), clinicians can stratify patients into lower- and higher-risk groups. For patients predicted to be at higher risk, intensified symptom monitoring and patient education on early lower urinary tract symptoms can be implemented, and supportive management (e.g., antispasmodics/analgesics or bladder mucosal protectants) may be considered to minimize treatment interruption. When clinically feasible, protocol optimization such as avoiding unnecessarily prolonged retention time may further reduce the risk of chemical cystitis. Conversely, patients at lower predicted risk may follow standard monitoring pathways.

Several limitations should be acknowledged. First, this was a single-center retrospective study, which may introduce selection bias and limit the generalizability of the findings. Second, due to the retrospective nature, information bias is possible because symptom severity and clinical interventions may not be uniformly documented across patients. Third, residual confounding cannot be excluded, and some potentially relevant variables (e.g., urinary pH, pre-instillation prophylactic medications, and other supportive measures) were not fully captured because of missing data. Fourth, although we defined chemical cystitis using standardized criteria and excluded bacterial infection based on urine culture, some degree of outcome misclassification may still occur given overlapping clinical presentations. Therefore, external validation in independent cohorts is warranted to confirm model transportability and clinical impact. Future multi-center prospective studies with external validation are needed to confirm transportability and evaluate clinical impact. Machine learning–based approaches have been increasingly used for bladder cancer risk stratification; nevertheless, an interpretable nomogram derived from a focused clinical question may be more readily implemented in routine practice (14). Beyond routinely available clinical variables, future models may incorporate additional data modalities (e.g., metabolic or molecular markers) to further refine risk stratification (15).

## Data Availability

The raw data supporting the conclusions of this article will be made available by the authors, without undue reservation.
